# The Cerebrovascular Reactivity Adjusted Fractional Amplitude of Low-Frequency Fluctuations Abnormalities in Middle Cerebral Artery Stenosis and Occlusive Disease

**DOI:** 10.1007/s12975-026-01430-z

**Published:** 2026-04-01

**Authors:** Liqing Zhang, Luoyu Wang, Xue Tang, Yidi Zhu, Rong Wang, Zhongxiang Ding

**Affiliations:** 1 Department of Radiology, Affiliated Hangzhou First People’s Hospital, School of Medicine, Westlake University, Hangzhou, 310006 China; 2 School of Biomedical Engineering, ShanghaiTech University, Shanghai, 201210 China; 3 Center for Cognition and Brain Disorders, The Affiliated Hospital of Hangzhou Normal University, Hangzhou, 311121 China; 4 School of Medical Imaging, Hangzhou Medical College, Hangzhou, 310006 China

**Keywords:** Resting-state functional magnetic resonance imaging, Fractional amplitude of low-frequency fluctuations, Cerebrovascular reactivity, Middle cerebral artery, Stenosis

## Abstract

This study investigated the variation characteristics of static fractional amplitude of low-frequency fluctuations (sfALFF) and dynamic fALFF (dfALFF) in Middle cerebral artery stenosis or occlusion(MCA-S) patients, and explored these two indicators alterations with cerebrovascular reactivity (CVR) correction. A total of 41 MCA-S patients and 50 matched controls underwent resting-state functional MRI and neuropsychological testing. Group differences in sfALFF/dfALFF were compared between the two groups, and the brain regions with differences in sfALFF/dfALFF with and without CVR correction were explored. Subsequently, partial correlation analysis was employed to evaluate the correlation between the abnormal brain regions and neuropsychological assessments. After CVR correction, MCA-S patients displayed increased sfALFF in Vermis_6, ipsilesional Cerebelum_8, Hippocampus, and other regions, alongside elevated dfALFF in Vermis_7, ipsilesional Hippocampus, and contralesional Cerebelum_8 (*P* < 0.001). No significant correlations were found between fALFF metrics and neuropsychological scores post-Bonferroni correction. Uncorrected analyses showed group differences in sfALFF/CVR within ipsilesional Occipital_Mid and Parietal_Inf, while CVR-adjusted results revealed changes in ipsilesional Cerebelum_8 and contralesional Caudate (*P* < 0.05). For dfALFF, uncorrected differences emerged in ipsilesional Postcentral, whereas CVR correction highlighted ipsilesional Temporal_Inf alterations. In summary, MCA-S patients exhibited abnormal neuronal activity associated with both sfALFF and dfALFF. With CVR correction, vascular confounding effects on the BOLD signal were partially mitigated, thereby enabling more accurate reflection of underlying neural activity alterations.

**Supplementary Information:**

The online version contains supplementary material available at 10.1007/s12975-026-01430-z.

## Introduction

Middle cerebral artery stenosis or occlusion(MCA-S) is a primary cause of ischemic stroke(IS) worldwide, and constitutes the most common type and an independent risk factor for stroke in Asian populations [1, 2]. The middle cerebral artery is frequently implicated in IS, often serving as the critical vessel involved in the condition’s pathogenesis. MCA-S induces significant hemodynamic disturbances [3], such as reduced cerebral blood flow (CBF) and a compromised oxygen extraction fraction, which ultimately precipitate ischemic brain injury and stroke. Chronic cerebral hypoperfusion due to intracranial arterial stenosis or occlusion is a key mechanism leading to vascular cognitive impairment and dementia [4, 5]. Moderate or severe vascular stenosis has been specifically linked to cognitive impairment, which can adversely affect patients’ daily life and behavior [6]. Given its high morbidity and association with poor neurological outcomes, elucidating the cerebral pathophysiology of MCA-S is essential for developing effective diagnostic and therapeutic strategies.

Resting-state functional magnetic resonance imaging (rs-fMRI) is a powerful tool for investigating brain activity in vivo, providing insights into the organization and dynamics of brain networks under normal and pathological conditions. A prominent analytical method in this field is the fractional Amplitude of Low-Frequency Fluctuation (fALFF) [7], which quantifies the relative amplitude of low-frequency fluctuations (0.01–0.08 Hz) in the blood oxygen level-dependent (BOLD) signal. Specifically, fALFF is calculated as the ratio of the power within this low-frequency band to the power across the entire detectable frequency spectrum of the Fourier-transformed time series. This normalization mitigates the influence of high-frequency physiological noise, such as respiratory and cardiac. Consequently, fALFF has become a widely utilized biomarker in neuropsychiatric research for conditions including stroke [8], depression [9], and Alzheimer’s disease [10](AD).

Although fALFF provides valuable information regarding the static characteristics of brain activity, it cannot reflect the dynamic, time-varying nature of neural processes. The brain is a highly adaptive and dynamic system that continuously adjusting its activity in response to internal and external stimuli [11, 12]. To better characterize these temporal fluctuations, the dynamic fractional amplitude of low-frequency fluctuations (dfALFF) was developed. dfALFF reflects the temporal variability in the fALFF values across different brain regions, offering further insight into how neural activity fluctuates over time [13, 14]. Combining this dynamic analysis with the static fALFF (sfALFF) provides a more comprehensive characterization of brain activity, elucidating both the spatial and temporal aspects of neural processes. However, the application of sfALFF and dfALFF in diseases involving intracranial vascular stenosis, such as MCA-S, remains unexplored.

However, calculating fALFF from BOLD signals may be confounded by hemodynamic influences. In fact, BOLD signals in fMRI do not directly measure neuronal activity but instead reflect hemodynamic changes mediated by neurovascular coupling, a process through which neuronal activation induces vascular responses to meet metabolic demands [15]. Consequently, the BOLD signal is modulated by both neural activity and vascular physiology, complicating the interpretation of fMRI data [16, 17]. Recent study have underscored the critical role of cerebrovascular reactivity (CVR)—the capacity of blood vessels to dilate in response to neuronal activation—in modulating BOLD signals [15]. CVR is a key determinant of the brain’s capacity to sustain sufficient blood flow during increased neural activity, and its impairment can affect the BOLD signal. Golestani et al. [18] showed a strong positive correlation between CVR and the rs-fMRI functional connectivity(FC) in healthy young individuals, demonstrating that CVR effects are both individual- and region-specific characteristics. This finding emphasizes the need to correct vascular factor in future studies, especially those focused on cerebrovascular diseases. In a study of patients with white matter hyperintensities (WMHs), Ni et al. [15] demonstrated that CVR significantly affected ALFF analysis. With CVR correction, it increased the sensitivity for detecting aberrant neural activity specifically associated with WMHs-related cognitive impairment. Their work highlighted the importance of CVR correction in rs-fMRI analysis, offers a new approach for eliminating vascular interference from neuronal activity signals. Wang et al. [19] comprehensively analyzed the potential effects of hemodynamic lag (LAG) and intrinsic CVR on rs-fMRI metrics. Their results indicated that at the local-scale, the amplitude of low-frequency fluctuations (ALFF) was mainly influenced by intrinsic CVR, while regional homogeneity (ReHo) was primarily affected by LAG. Correcting for CVR optimized the detection of post-stroke cerebral functional abnormalities. Due to the vascular etiology of MCA-S, the impact of altered vascular physiology should be considered when assessing neural activity in these patients. Crucially, the application of CVR correction to dfALFF has not been explored in prior research. Therefore, integrating CVR correction into fALFF analysis is expected to provide more precise and reliable results of brain function in MCA-S.

This study focused on patients with MCA-S, a distinct model of chronic hemodynamic impairment, and employed rs-fMRI to systematically investigate CVR-corrected fALFF metrics, including sfALFF and dfALFF. The study comprised two parts: first, we investigated the inter-group differences of sfALFF and dfALFF between MCA-S patients and healthy controls, and assessed their correlation with clinical indicators. Second we explored the feasibility of applying CVR-corrected sfALFF and dfALFF in patients with MCA-S. The study aimed to dissociate vascular artifacts from neural activity signals, to provide more reliable neuroimaging biomarkers for the accurate assessment of neurofunctional abnormalities in MCA-S patients.

## Materials and Methods

### Participants

**Fig. 1:**
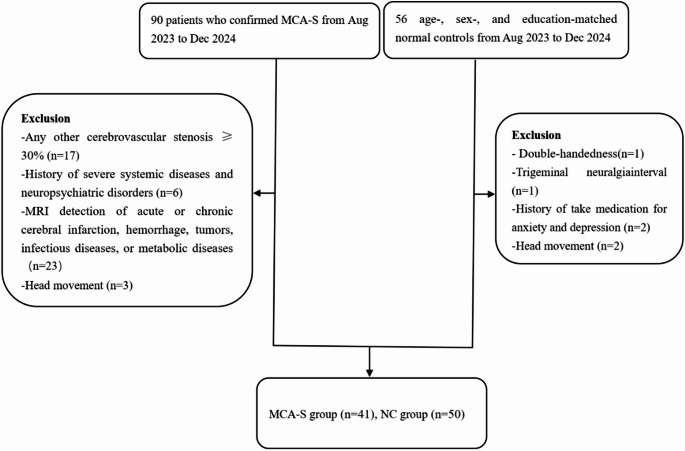
Flowchart of the present study. MCA-S, Middle cerebral artery stenosis; NC, Normal control

The study was approved by the Ethics Committee of Hangzhou First People’s Hospital (IIT-20230822-0181-01), and all participants signed informed consent forms. Between September 2023 and December 2024, a total of 90 patients with middle cerebral artery stenosis were recruited, along with 56 healthy subjects matched for age, gender, and education level from the community as the normal control group (NC group). The inclusion criteria were as follows: (1) age between 18 and 85 years; (2) unilateral middle cerebral artery stenosis (≥ 50%); (3) no significant abnormalities detected by diffusion weighted imaging (DWI); (4) right-handedness. Exclusion criteria were: (a) any other cerebrovascular stenosis ≥ 30%, (b) history of severe systemic diseases, neuropsychiatric diseases, or neurodegenerative diseases, (c) MRI showing acute or chronic cerebral infarction, hemorrhage, tumors, infectious diseases, or metabolic diseases, (d) incomplete clinical data or non-standard images, (e) excessive head movement, and (f) relative and absolute contraindications for MRI examination. Therefore, the final study cohort consisted of 41 patients with middle cerebral artery stenosis and 50 normal subjects (Fig. 1).

### Neuropsychological Evaluation

Specialized physicians conducted clinical psychological assessments for all subjects. The Mini-Mental State Examination (MMSE) was utilized to evaluate cognitive function, while the Self-Rating Depression Scale (SDS) and Self-Rating Anxiety Scale (SAS) assessed depressive and anxiety symptoms, respectively. Additionally, the Pittsburgh Sleep Quality Index (PSQI) was employed to assess sleep quality. According to Chinese norms [20], the cutoff score for the SAS is 50, and the cutoff score for the SDS is 53. The PSQI [21] provides an overall measure of sleep quality, where a score below 5 indicates good sleep quality, 5–8 indicates poor sleep quality and a score exceeding 8 suggests the presence of insomnia or other severe sleep disturbances.

### MRI Data Acquisition

The MRI data were acquired with a 3.0T magnetic resonance scanner (Premier MR750, GE Healthcare, Wauke-sha, WI, USA) and a 48-channel phased array head coil. During the scanning, participants were positioned in a supine position with their heads placed first into the scanner. They were instructed to keep their eyes closed and maintain calm breathing while remaining awake [19, 22]. To minimize head movement, foam pads were placed on both sides of the head for stabilization. All subjects wore earplugs to reduce noise interference and protect their hearing. The scanning parameters were as follows:

1. T1-weighted 3D fast spoiled gradient-recalled (3D-T1WI): repetition time (TR) = 2616 ms, echo time (TE) = 2.9 ms, inversion time (TI) = 1000 ms, flip angle (FA) = 8°, matrix size = 256 × 256, field of view (FOV) = 256 mm × 256 mm, voxel size = 1 mm × 1 mm × 1 mm, slice thickness/gap = 1/1 mm, slice = 192.
2. T2 weighted Imaging(T2WI): TR = 4753 ms, TE = 120 ms, matrix size = 352 × 352, FOV = 240 mm × 240 mm, voxel size = 0.6 mm × 0.6 mm × 6.0 mm. slice thickness/gap = 6/1 mm, slice = 20.
3. T2 fluid attenuated inversion recovery(FLAIR): TR = 9000 ms, TE = 100 ms, matrix size = 288 × 256, FOV = 240 mm × 240 mm, voxel size = 0.8 mm × 0.9 mm × 6.0 mm, slice thickness/gap = 6/1 mm, slice = 20.
4. axial diffusion-weighted imaging (DWI): TR = 2115 ms, TE = 62 ms, matrix size = 160 × 168, FOV = 240 mm × 240 mm, voxel size = 1.5 mm × 1.4 mm × 6.0 mm, b value = 1000 s/mm^2^, slice thickness/gap = 6/1 mm, slice = 40.
5. axial rs-fMRI: TE = 30 ms, FA = 56°, matrix size = 80 × 80, FOV = 240 mm × 240 mm, voxel size = 3 mm × 3 mm × 3 mm, volumes = 600, slice thickness = 3 mm, slice = 52, and parallel acceleration = 4, phase encoding PA direction, and the acquisition duration was 8 min. To perform magnetic susceptibility correction, we also acquired echo-planar imaging data with reversed phase encoding directions using the same acquisition parameters.

### Analysis of White Matter Hyperintensity (WMH)

WMH was assessed independently by two radiologists with over 10 years of experience in a double-blind manner. In case of disagreement between the raters, consensus was reached through discussion. Fazekas grading was performed based on the Fazekas [23] classification system, where the total score was obtained by summing up the scores for periventricular white matter hyperintensity and deep white matter hyperintensity. For periventricular hyperintensity scoring: 0 points for no lesions; 1 point for cap-like or pencil-thin layered lesions; 2 points for lesions presenting as a smooth halo; and 3 points for irregular periventricular hyperintensity extending into the deep white matter. According to the total score, Fazekas grade 0 was 0; Fazekas grade 1: 1 ~ 2 points; Fazekas 2: 3 ~ 4 points; Fazekas grade 3: 5 ~ 6 points.

### Visualization of the Middle Cerebral Artery Territory

The middle cerebral artery territory [24, 25] segmentation was manually delineated by one radiologists with more than 10 years of experience in neurological imaging by ITK-SNAP (version 3.6.0, http://itksnap.org) in 3D-T1WI sequence.

## MRI Data Processing

### Image Flipping

To standardize the lesion side, following previous study, the 3D-T1WI structural and BOLD images of 23 patients with right MCA-S were horizontally flipped, effectively flipping the right side to the left. This defined the left side as the ipsilesional side and the right side as the contralesional side. This standardization facilitated subsequent statistical analysis and functional investigations.

### CVR Calculation

According to previous research [19, 26], the calculation of CVR was performed using the FSL, ANTs and SPM12 toolboxes. Following the validated method by Liu et al. [27], the reference timecourse was derived from the whole-brain global mean signal. Following these steps: (1) Discarding the first 10 time points to ensure subjects’ adaptation to the scanning environment and stability of the MRI signal; (2) Head motion correction: we utilized the Friston-24 parameter model to regress out head motion effects. High-motion volumes (framewise displacement > 0.5 mm) were scrutinized/scrubbed to ensure the “global signal” reflected vascular fluctuations rather than movement artifacts. And excluding subjects with head motion displacement >3 mm, rotation > 3°, or mean framewise displacement (FD) > 0.5; (3) Correcting spatial distortions and susceptible artifacts; (4) Aligning with structural MRI using tissue boundary maps for precise functional localization; (5) Applying a one-time resampling in each subject’s native space, incorporating all aforementioned transformations to avoid repeated interpolation and signal smoothing. (6) Spatial normalization to the MNI standard space and resampling to 3 × 3 × 3 mm^3^ voxels. (7) Spatial smoothing was applied using a 6 mm FWHM isotropic Gaussian kernel to reduce noise and improve the signal-to-noise ratio. Furthermore, signals from white matter and cerebrospinal fluid were regressed out during preprocessing to reduce non-neuronal/non-vascular noise contributions. (8) Linear detrending and band-pass filtering (0.02–0.04 Hz) were carried out. Based on previous research by Liu et al. [27] global BOLD signal fluctuations within this frequency range are most correlated with natural arterial CO2 variations during spontaneous breathing. (9) A voxel-wise analysis was conducted using the general linear model (GLM), with the reference time course as the independent variable. The results were normalized by dividing by the global mean, yielding the relative CVR index for each voxel. Figure S1 showed the averaged CVR maps obtained from the MCA-S and NC groups.

### sfALFF and dfALFF Calculation

The calculation of sfALFF and dfALFF was performed using the FSL, ANTs and Data Processing & Analysis of Brain Imaging (DPABI) software. Following these steps: (1) As mentioned above, preprocessing involved discarding initial time points, motion correction, distortion correction, structural alignment, and one-time resampling for functional localization. (2) Utilizing ICA AROMA for noise identification, primarily to detect motion-related artifacts. (3) Conducting a one-time regression on identified noise components, Friston 24 motion parameters, linear trends, and signals from white matter and cerebrospinal fluid to purge confounds from the data. (4) Normalizing based on tissues segmented by ANTs, aligning individual brain images with MNI space according to tissue segmentation, and then resampling to a 3 × 3 × 3 mm^3^ voxels size to ensure anatomical consistency across subjects for accurate group analysis. (5) Fast Fourier Transform (FFT) was applied to the time series of each voxel, converting the BOLD signal from the time domain to the frequency domain to obtain the power spectrum. (6) The square root of each frequency domain power spectrum was calculated, and the average square root within the frequency range of 0.01–0.08 Hz was taken as the sfALFF/dfALFF value. (7) The sliding window method was used to calculate dfALFF, as it is highly sensitive to temporal variations and can assess the variability of whole-brain metrics [28]. Window length and step size are two critical parameters in this approach [29]. An optimal window length must be short enough to detect transient signals, yet long enough to analyze the lowest frequency of interest [30]. Excessively short windows can introduce spurious fluctuations and undermine the robustness of subsequent calculations, whereas overly long windows may obscure dynamic features [31]. The sfALFF/dfALFF value of each voxel was then normalized by dividing it by the mean sfALFF/dfALFF value of the whole brain. To ensure the reliability of the results, dynamic methods with sliding window length of 50TR (40s) with moving step of 5TR (4s), as well as a sliding window length of 125TR (100s) and a moving step of 5TR (4s) were used for validation. (8) Standard deviations of sfALFF/dfALFF for all voxels in all time windows were calculated for each subject to evaluate temporal variability. (9) Finally, spatial smoothing was performed using a Gaussian kernel of 6 mm FWHM.

### Statistical Analysis

Statistical analysis of clinical data and scales was performed using SPSS version 26.0 (SPSS Inc., Chicago, IL, USA), GraphPad Prism 9. Cohen’s Kappa test was used to assess the consistency of WMH grading parameters measured by two observers. Enumeration data were expressed as rates or proportions, while measurement data conforming to a normal distribution were described using mean ± standard deviation ($\:\stackrel{-}{x}\pm\:\mathrm{s}$). Demographic information, clinical indicators, and neuropsychological test scores were compared between the MCA-S group and the NC group. Independent sample *t*-tests were used to compare the means of measurement data, while chi-square (*χ2*) tests were used for enumeration data.

In the DPABI software of MATLAB, gray matter templates were used to remove interference from white matter, cerebrospinal fluid, etc. Age, gender, and education level were included as covariates. A mass-univariate Analysis of Covariance (ANCOVA) operating within a General Linear Model (GLM) framework were performed to analyze and compare sfALFF, dfALFF and corresponding CVR value in the classic frequency band (0.01–0.08 Hz and 0.02–0.04 Hz) between the two groups. Subsequently, subject-specific spatial CVR maps were entered as voxel-dependent covariates in a mass-univariate GLM to obtain the CVR-corrected sfALFF/dfALFF. If there were statistical differences between the two groups, multiple comparison corrections were performed using Gaussian Random Field (GRF) theory (voxel *P* < 0.001, cluster *P* < 0.05). For any brain regions showing differences between groups, partial correlation analysis was used to evaluate the correlation between the mean values of these regions and the MMSE, MoCA, SAS/SDS, and PSQI scores of the subjects. Statistical significance was determined using a Bonferroni-corrected threshold of *P* < 0.01 (0.05/5). To explicitly evaluate the independent impact of hypertension on the outcomes, the GLM ANCOVA was repeated as a secondary sensitivity analysis with age, sex, educational level, and hypertension status included as nuisance covariates.

## Results

### Demographics and Clinical Characteristics

Table 1 presents the demographic, clinical characteristics, and neuropsychological assessments of the participants. This study included 41 patients with middle cerebral artery stenosis (MCA-S) and 50 normal controls matched for age, gender, and years of education. All MCA-S patients had unilateral onset (23 on the right side and 18 on the left side). The proportion of hypertension in the MCA-S group was higher than that in the NC group, with a significant difference between the two groups (*P* = 0.010). A supplementary telephone follow-up with the hypertensive participants regarding their pharmacological management during the study period.The vast majority of hypertensive patients in both two groups were on regular antihypertensive regimens with well-controlled blood pressure. There was no statistically significant difference between the two groups in the proportion of subjects with well-controlled blood pressure (*P* = 0.263). The consistency analysis between two observers for Fazekas grading yielded a Cohen’s kappa of 0.79. There was no significant difference in white matter hyperintensity grading between the two groups. Compared to healthy controls, patients in the MCA-S group had lower scores on both MMSE and MoCA, with a statistically significant difference in MMSE scores between the two groups (*P* = 0.003). However, there were no statistically significant differences in anxiety, depression, or sleep status between the two groups.

**Table 1:** Demographic and clinical characteristics of MCA-S and NC groups

	MCA-S (*n* = 41)	NC (*n* = 50)	χ2/t	*P* value
Sex				
Male	25	30	0.009	0.925
Female	16	20		
Age(years)	58.51 ± 14.82	59.38 ± 12.25	2.081	0.153
Education	6.80 ± 4.11	8.30 ± 5.23	1.584	0.211
Fazekas grade			2.313	0.128
0–1	29	42		
2–3	12	8		
BMI	24.85 ± 2.52	23.71 ± 2.68	0.001	0.976
Smoking, n(%)			0.999	0.318
Yes	19(46%)	18(36%)		
No	22(54%)	32(64%)		
Drinking, n(%)			0.400	0.527
Yes	14(34%)	14(28%)		
No	27(66%)	36(72%)		
Hypertension, n(%)			6.613	0.010
Yes	32(78%)	26(52%)		
No	9(22%)	24(48%)		
Hyperlipidemia, n(%)			2.653	0.103
Yes	20(49%)	16(32%)		
No	21(51%)	34(68%)		
Diabetes, n(%)			3.110	0.078
Yes	15(37%)	10(20%)		
No	26(63%)	40(80%)		
Coronary disease, n(%)			0.583	0.445
Yes	5(12%)	9(18%)		
No	36(88%)	41(82%)		
MMSE	26.46 ± 4.94	28.02 ± 2.32	9.307	0.003
MoCA	23.86 ± 6.38	24.87 ± 4.40	2,535	0.115
SAS	30.16 ± 6.14	29.26 ± 5.37	1.320	0.254
SDS	33.32 ± 7.65	31.40 ± 5.73	2.418	0.124
PSQI	6.51 ± 4.15	5.60 ± 3.57	1.490	0.226

*MCA-S *Middle cerebral artery stenosis, *MMSE *Mini-mental state examination, *MoCA* Montreal cognitive assessment, *NC *Normal control, *PSQI *Pittsburgh sleep quality index, *SAS* Self rating anxiety seale,* SDS *Self rating depression seale

### Differences Between Groups in sfALFF and dfALFF with CVR Correction

**Fig. 2:**
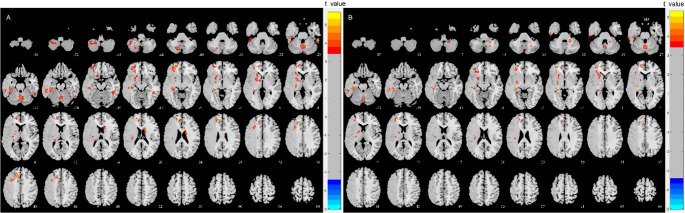
Altered brain regions in sfALFF and dfALFF MCA-S patients compared to NC with CVR correction combined visualization of the middle cerebral artery territory. (**A**) sfALFF, (**B**) dfALFF. CVR, Cerebrovascular reactivity; dfALFF, Dynamic amplitude of low frequency fluctuation; MCA-S, Middle cerebral artery stenosis; NC, Normal control; sfALFF, Static amplitude of low frequency fluctuation

Compared with NC group, MCA-S patients showed increased sfALFF values with CVR correction in Cerebellar Vermis VI (Vermis_6) [32], ipsilesional Cerebellum Crus VIII‌(Cerebelum_8), Hippocampus, ‌Superior Frontal gyrus (Frontal_Sup), Lenticular nucleus putamen(Putamen), Insula, contralesional Inferior Temporal gyrus (Temporal_Inf), and Caudate nucleus (Caudate) (*P* < 0.001, cluster *P* < 0.05) (Table 2; Fig. 2**)**. Additionally, MCA-S patients exhibited increased dfALFF values with CVR correction in Cerebellar Vermis VII (Vermis_7), ipsilesional Hippocampus, Frontal_Sup, Putamen, Temporal_Inf, and contralesional Cerebelum_8 (*P* < 0.001, cluster *P* < 0.05) (Table 2; Fig. 2). When hypertension status was incorporated into the model, the spatial distribution and statistical significance of the results remained virtually unchanged, confirming that the functional alterations are not primarily driven by systemic hypertensive effects (Table S1, Figure S2).

**Table 2:** Brain regions of significant differences in sfALFF, dfALFF with CVR correction between MCA-S and NC groups

Brain regions		Peak MNI coordinates			Cluster size(voxels)	Peak intensity	*P*
		X	Y	Z			
sfALFF	Hippocampus_IL	-33	-30	-6	30	5.0800	< 0.001
	Vermis_6	-3	-63	-24	30	4.4864	< 0.001
	Frontal_Sup_IL	-18	39	27	92	5.6889	< 0.001
	Putamen_IL	-24	6	0	48	4.6957	< 0.001
	Cerebelum_8_ IL	-39	-42	-45	52	4.0531	< 0.001
	Temporal_Inf_CON	48	-39	-15	39	4.2651	< 0.001
	Insula_IL	-21	-30	15	12	4.5074	< 0.001
	Caudate_CON	9	9	21	8	4.9555	< 0.001
dfALFF	Vermis_7	-3	-66	-27	8	4.4277	< 0.001
	Cerebelum_8_CON	21	-48	-42	12	4.6255	< 0.001
	Hippocampus_IL	-33	-33	-3	23	5.1448	< 0.001
	Putamen_IL	-30	-18	6	28	4.8693	< 0.001
	Frontal_Sup_IL	-15	57	6	39	5.2348	< 0.001
	Temporal_Inf_IL	-57	-33	-18	41	5.021	< 0.001

*Caudate* Caudate nucleus, *Cerebelum_8* Cerebellum Crus VIII‌, *CON* Contralateral, *CVR *Cerebrovascular reactivity, *dfALFF* Dynamic amplitude of low frequency fluctuation, *Frontal_Sup* Superior Frontal gyrus, *IL* ipsilateral, *MCA-S* Middle cerebral artery stenosis, *MNI* Montreal neurological institute, *NC* Normal control, *Putamen* Lenticular nucleus putamen, *sfALFF* Static amplitude of low frequency fluctuation, *Temporal_Inf* Inferior Temporal gyrus, *Vermis_6* Cerebellar Vermis VI, *Vermis_7* Cerebellar Vermis VII

### Correlation Analysis Between sfALFF, dfALFF, and Scales with CVR Correction

After Bonferroni’s correction, partial correlation analysis indicated no significant correlation between sfALFF, dfALFF, and MMSE, MoCA, SAS/SDS, or PSQI with CVR correction (*P* > 0.05) (Table 3).

**Table 3:** The correlation coefficients and the corresponding *P* values between sfALFF, dfALFF of inconsistent brain regions and scales with CVR correction

Brain regions			MMSE	MoCA	SAS	SDS	PSQI
sALFF	Caudate _CON	*r*	-0.078	-0.05	0.066	0.214	0.140
		*P*	0.648	0.771	0.698	0.204	0.409
	Cerebelum_8_IL	*r*	0.030	-0.082	0.093	0.158	0.190
		*P*	0.858	0.629	0.585	0.351	0.261
	Insula_IL	*r*	-0.036	-0.006	0.090	0.244	0.146
		*P*	0.833	0.973	0.595	0.146	0.389
	Temporal_Inf_CON	*r*	-0.047	-0.006	-0.008	0.097	0.129
		*P*	0.783	0.971	0.965	0.567	0.446
	Vermis_6	*r*	-0.044	-0.056	0.040	0.212	0.144
		*P*	0.796	0.743	0.812	0.208	0.395
	Frontal_Sup_IL	*r*	0.242	0.209	-0.236	-0.05	-0.161
		*P*	0.148	0.214	0.160	0.770	0.342
	Hippocampus_IL	*r*	0.040	-0.023	0.035	0.139	0.166
		*P*	0.816	0.891	0.835	0.412	0.325
	Putamen_IL	*r*	-0.151	-0.099	0.079	0.145	0.122
		*P*	0.371	0.559	0.641	0.393	0.471
dALFF	Temporal_Inf_IL	*r*	0.026	-0.053	0.054	0.111	0.161
		*P*	0.880	0.755	0.753	0.511	0.342
	Cerebelum_8_CON	*r*	-0.048	-0.115	0.009	0.169	0.062
		*P*	0.779	0.497	0.956	0.319	0.717
	Frontal_Sup_IL	*r*	0.135	0.162	-0.24	-0.029	-0.138
		*P*	0.424	0.337	0.153	0.866	0.416
	Hippocampus_IL	*r*	0.003	-0.024	0.112	0.218	0.320
		*P*	0.984	0.889	0.510	0.196	0.054
	Putamen_IL	*r*	0.029	0.053	0.159	0.277	0.118
		*P*	0.866	0.754	0.348	0.097	0.485
	Vermis_7	*r*	0.060	0.014	0.057	0.240	0.150
		*P*	0.723	0.933	0.739	0.153	0.375

*Caudate* Caudate nucleus, *Cerebelum_8* Cerebellum Crus VIII‌, *CON* Contralateral, *CVR *Cerebrovascular reactivity, *dfALFF* Dynamic amplitude of low frequency fluctuation, *Frontal_Sup* Superior Frontal gyrus, *IL* ipsilateral, *MMSE* Mini-mental state examination, *MoCA *Montreal cognitive assessment, *PSQI* Pittsburgh sleep quality index, *Putamen* Lenticular nucleus putamen; *SAS* Self rating anxiety seale, *SDS *Self rating depression seale, *sfALFF* Static amplitude of low frequency fluctuation, *Temporal_Inf* Inferior Temporal gyrus, *Vermis_6* Cerebellar Vermis VI, *Vermis_7* Cerebellar Vermis VII

### Differences Between Groups in sfALFF and dfALFF Without CVR Correction

**Fig. 3:**
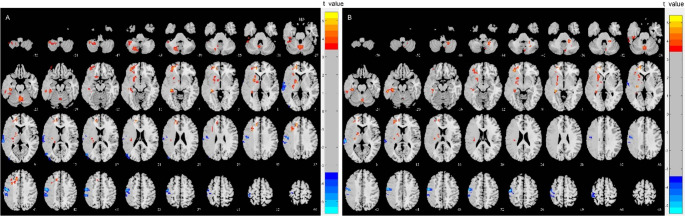
Altered brain regions in sfALFF and dfALFF MCA-S patients compared to NC without CVR correction combined visualization of the middle cerebral artery territory. (**A**) sfALFF, (**B**) dfALFF. CVR, Cerebrovascular reactivity; dfALFF, Dynamic amplitude of low frequency fluctuation; MCA-S, Middle cerebral artery stenosis; NC, Normal control; sfALFF, Static amplitude of low frequency fluctuation

Compared with NC group, MCA-S patients had increased static fALFF values without CVR correction in Vermis_6, ipsilesional Hippocampus, Frontal_Sup, and Putamen (*P* < 0.001, cluster *P* < 0.05). However, they also displayed decreased sfALFF values without CVR correction in ipsilesional Middle Occipital gyrus (Occipital_Mid), Inferior Parietal gyrus (Parietal_Inf), and Postcentral gyrus (Postcentral) (*P* < 0.001, cluster *P* < 0.05) (Table 4; Fig. 3). MCA-S patients had increased dfALFF values without CVR correction in Vermis_7, ipsilesional Hippocampus, Frontal_Sup, Putamen, Superior temporal gyrus(Temporal_Sup), Postcentral, and contralesional Cerebelum_8 (*P* < 0.001, cluster *P* < 0.05) (Table 4; Fig. 3). When hypertension status was incorporated into the model, the spatial distribution and statistical significance of the results remained virtually unchanged, confirming that the functional alterations are not primarily driven by systemic hypertensive effects (Table S2, Figure S3).

**Table 4:** Brain regions of significant differences in sfALFF, dfALFF without CVR correction between MCA-S and NC groups

	Brain regions	Peak MNI coordinates			Cluster size(voxels)	Peak intensity	*P*
		X	Y	Z			
sfALFF	Hippocampus_IL	-33	-30	-6	33	5.1174	< 0.001
	Vermis_6	-3	-63	-24	32	4.4727	< 0.001
	Frontal_ Sup _IL	-18	39	27	86	5.4792	< 0.001
	Putamen_IL	-24	6	0	66	4.9329	< 0.001
	Occipital_Mid_IL	-51	-72	15	9	-5.6881	< 0.001
	Parietal_Inf_IL	-54	-33	48	48	-4.671	< 0.001
	Postcentral_IL	-51	-12	42	217	-5.5987	< 0.001
dfALFF	Vermis_7	-3	-66	-27	9	4.4935	< 0.001
	Cerebelum_8_CON	21	-48	-42	16	4.6569	< 0.001
	Hippocampus_IL	-33	-33	-3	27	5.195	< 0.001
	Putamen_IL	-27	-18	6	59	4.8754	< 0.001
	Frontal_Sup_IL	-15	57	6	17	5.2501	< 0.001
	Temporal_Sup_IL	-63	-27	9	45	-5.1535	< 0.001
	Postcentral_IL	-54	-12	45	158	-5.4613	< 0.001

*Cerebelum_8* Cerebellum Crus VIII‌, *CON* Contralateral, *CVR *Cerebrovascular reactivity, *dfALFF* Dynamic amplitude of low frequency fluctuation, *Frontal_Sup* Superior Frontal gyrus, *IL* ipsilateral, *MCA-S* Middle cerebral artery stenosis, *MNI* Montreal neurological institute, *NC* Normal control, *Occipital_Mid* Middle Occipital gyrus, *Parietal_Inf *Inferior Parietal gyrus, *Postcentral* Postcentral gyrus, *Putamen* Lenticular nucleus putamen, *sfALFF *Static amplitude of low frequency fluctuation, *Temporal_Inf* Inferior Temporal gyrus, *Temporal_Sup *Superior temporal gyru, *Vermis_6* Cerebellar Vermis VI, *Vermis_7* Cerebellar Vermis VII

### Comparison of Brain Regions With Differences in sfALFF and dfALFF Between the Two Groups With and Without CVR Correction

**Fig. 4:**
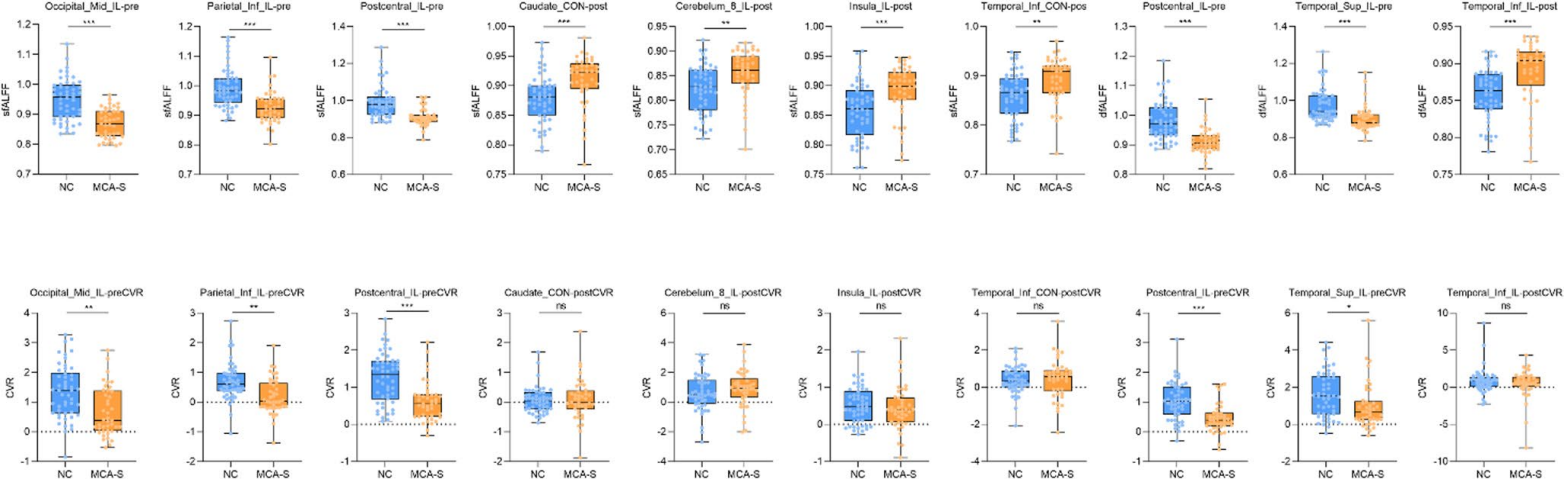
Differences in sfALFF, dfALFF and corresponding CVR between MCA-S and NC groups. The first row of figures presents the between-group comparisons of sfALFF and dsfALFF in various differential brain regions, while the second row displays the corresponding between-group comparisons of CVR values in these brain regions. Post hoc comparisons of *t* test. (voxel *P* < 0.001, cluster *P* < 0.05, GRF correction.*** indicates a significant level of *P* < 0.001, ** indicates a significant level of *P* < 0.01, *indicates a significant level of 0.01 < *P* < 0.05, ns indicates a significant level of *P* > 0.05 Bonferroni correction). Caudate, Caudate nucleus; Cerebelum_8, Cerebellum Crus VIII‌; CON, Contralateral; CVR, Cerebrovascular reactivity; Frontal_Sup, Superior Frontal gyrus; IL, ipsilateral; MCA-S, Middle cerebral artery stenosis; NC, Normal control; Occipital_Mid, Middle Occipital gyrus; Parietal_Inf, Inferior Parietal gyrus; post, with CVR correction; Postcentral, Postcentral gyrus; Putamen, Lenticular nucleus putamen; pre, without CVR correction; Temporal_Inf, Inferior Temporal gyrus; Temporal_Sup, Superior temporal gyru; Vermis_6, Cerebellar Vermis VI; Vermis_7, Cerebellar Vermis VII

 The results revealed significant differences between groups in sfALFF values and CVR values without CVR correction in ipsilesional Occipital_Mid, Parietal_Inf, and Postcentral (*P* < 0.05). Significant differences in sfALFF values with CVR correction were observed in ipsilesional Cerebelum_8, Insula_L, contralesional Temporal_Inf, and Caudate (*P* < 0.05), while no significant differences in CVR values were found with correction (*P* > 0.05). For dfALFF, significant differences between groups were observed in ipsilesional Postcentral, Temporal_Sup before CVR correction, and in ipsilesional Temporal_Inf with CVR correction (*P* < 0.05). However, there were no significant differences in CVR values with correction (*P* > 0.05). Similar results were obtained after FDR corrected. (Table 5; Fig. 4).

**Table 5:** Comparison of sfALFF, dfALFF between MCA-S and NC groups without and with CVR correction in inconsistent brain regions

Brain regions		MCA-S (*n* = 41)	NC (*n* = 50)	t	*P* value	FDR corrected *P* value
sfALFF	Occipital_Mid_IL-pre	0.869 ± 0.049	0.949 ± 0.070	6.087	0.000	0.000
	Parietal_Inf_IL-pre	0.928 ± 0.055	0.992 ± 0.065	5.082	0.000	0.000
	Postcentral_IL-pre	0.911 ± 0.047	0.993 ± 0.089	5.433	0.000	0.000
	Caudate_CON-post	0.909 ± 0.043	0.878 ± 0.041	-3.878	0.000	0.000
	Cerebelum_8_IL-post	0.854 ± 0.048	0.824 ± 0.049	-3.491	0.001	0.001
	Insula_IL-post	0.891 ± 0.044	0.857 ± 0.047	-3.907	0.000	0.000
	Temporal_Inf_CON-post	0.893 ± 0.046	0.859 ± 0.049	-3.600	0.001	0.001
	Occipital_Mid_IL-preCVR	0.664 ± 0.821	1.358 ± 0.904	3.534	0.001	0.001
	Parietal_Inf_IL-preCVR	0.190 ± 0.612	0.686 ± 0.635	3.669	0.000	0.001
	Postcentral_IL-preCVR	0.616 ± 0.537	1.253 ± 0.684	4.621	0.000	0.000
	Caudate_CON-postCVR	0.086 ± 0.697	0.067 ± 0.443	-0.009	0.993	0.993
	Cerebelum_8_IL-postCVR	0.911 ± 1.234	0.650 ± 1.232	-1.069	0.288	0.339
	Insula_IL-postCVR	0.428 ± 0.585	0.522 ± 0.496	0.986	0.327	0.363
	Temporal_Inf_CON-postCVR	0.456 ± 1.068	0.381 ± 0.734	-0.268	0.789	0.831
dfALFF	Postcentral _IL-pre	0.912 ± 0.042	0.981 ± 0.042	5.917	0.000	0.000
	Temporal_Sup_IL-pre	0.981 ± 0.067	0.972 ± 0.090	4.062	0.000	0.000
	Temporal_Inf_IL-post	0.889 ± 0.042	0.858 ± 0.035	-4.172	0.000	0.000
	Postcentral_IL-preCVR	0.448 ± 0.483	1.029 ± 0.628	4.691	0.000	0.000
	Temporal_Sup_IL-preCVR	0.986 ± 1.241	1.609 ± 1.237	2.368	0.020	0.027
	Temporal_Inf_IL-postCVR	0.454 ± 2.017	0.839 ± 1.674	1.290	0.201	0.251

*Caudate* Caudate nucleus, *Cerebelum_8* Cerebellum Crus VIII‌, *CON* Contralateral, *CVR *Cerebrovascular reactivity, *dfALFF* Dynamic amplitude of low frequency fluctuation, *FDR *False discovery rate,* Frontal_Sup *Superior Frontal gyrus,* IL* ipsilateral, *MCA-S* Middle cerebral artery stenosis, *MNI* Montreal neurological institute, *NC* Normal control, *Occipital_Mid* Middle Occipital gyrus, *Parietal_Inf* Inferior Parietal gyrus, *post* with CVR correction, *Postcentral* Postcentral gyrus, *Putamen* Lenticular nucleus putamen, *pre *without CVR correction, *sfALFF* Static amplitude of low frequency fluctuation, *Temporal_Inf* Inferior Temporal gyrus, *Temporal_Sup* Superior temporal gyru, *Vermis_6* Cerebellar Vermis VI, *Vermis_7* Cerebellar Vermis VII

## Discussion

This study employed both sfALFF and dfALFF analyses to investigate the alterations in spontaneous neural activity in patients with MCA-S. Additionally, this study shed new light on the potential influence of CVR on fALFF, including the impact on the dynamic functional metric (dfALFF). Compared to the NC group, MCA-S group exhibited increased CVR-corrected sfALFF values in Vermis_6, ipsilesional Cerebelum_8, Hippocampus, Frontal_Sup, Putamen, Insula, as well as the contralesional Temporal_Inf and Caudate. Similarly, elevated CVR-corrected dfALFF values were observed in Vermis_7, the ipsilesional Hippocampus, Frontal_Sup, Putamen, Temporal_Inf, and the contralesional Cerebelum_8. To further investigate the potential influence of CVR on both sfALFF and dfALFF measurements in MCA-S patients, the differences pre- and post-CVR correction were analyzed. Analyses without CVR correction revealed significant intergroup differences in both sfALFF values and CVR values within the ipsilesional Occipital_Mid, Parietal_Inf, and Postcentral. Significant intergroup differences were also detected in without CVR correction dfALFF values and CVR values in the ipsilesional Postcentral and Temporal_Sup. Our findings demonstrate that CVR significantly influences fALFF analyses. Moreover, with CVR correction results appear to provide a more accurately representation of disease-related aberrant neural activity in MCA-S patients, potentially allowing for a more nuanced understanding of hypoperfusion and CVR deficits on neurovascular coupling.

In our study, the MCA-S group exhibited a higher prevalence of hypertension. Previous studies reflected the hypertension and antihypertensive medications can alter vascular tone and BOLD signal characteristics. To address the effect of hypertension, we conducted re-analysis of hypertension as an additional covariate. The results showed that the main effects and spatial patterns of the fALFF and dfALFF abnormalities remained statistically significant and virtually unchanged after this adjustment. This stability in our results demonstrates that the observed neurovascular deficits are robust and are not driven solely by the presence of diagnosis-based hypertension. Furthermore, we conducted a supplementary telephone follow-up with the hypertensive participants regarding their pharmacological management during the study period. The data indicated that the vast majority of hypertensive patients in both the stenosis and control groups were on regular antihypertensive regimens with well-controlled blood pressure. Crucially, the proportion of subjects with well-controlled blood pressure did not differ significantly between the two groups, which suggested that the confounding effect of medication disparity or acute blood pressure fluctuation was small. Although hypertension typically promotes systemic arteriosclerosis that affects the cerebral vasculature globally and bilaterally, our results demonstrated a lateralized pattern of neurovascular decoupling predominantly in the hemisphere ipsilateral to the MCA-S. This spatial specificity strongly supports that the observed deficits are primarily from the focal hemodynamic compromise of the stenotic MCA, rather than from the generalized systemic vascular disease.

MCA-S patients serve as a unique model of chronic hemodynamic compromise without the confounding effects of large-scale tissue necrosis seen in stroke [19]. Our findings indicated that CVR correction reveals additional fALFF-altered brain regions. This suggests that accounting for vascular factors may provide a more specific assessment of neural activity alterations, thereby reducing potential confounding effects of CVR deficits on the interpretation of BOLD signals. Regions such as the hippocampus, superior frontal gyrus, insula, and inferior temporal gyrus—key nodes within the default mode network (DMN)—are associated with cognitive decline [33, 34]. Clinical data analysis indicated that patients with MCA-S patients had lower MMSE and MoCA scores than NC group, along with elevated sfALFF values in the hippocampus, superior frontal gyrus, insula, and inferior temporal gyrus. However, no correlation was observed between these brain regions and cognitive scale scores. This finding may hint at a compensatory mechanism of neural activity, a possibility that warrants further validation. The hippocampus is a critical region within the central nervous system involved in learning and memory consolidation. Huang et al. [35] reported increased fALFF in the hippocampus of patients with bipolar disorder, yet no association with cognitive function was identified, which consistent with our findings. The insula plays a pivotal role in cognition, emotion, autonomic regulation, and sensory processing. Xu et al. [36]. found significantly lower fALFF in the insula in mild cognitive impairment (MCI) compared to those with AD, alongside distinct functional connectivity patterns (e.g., hippocampus-inferior temporal gyrus, hippocampus-anterior cingulate cortex) in the MCI group. A meta-analysis by Zhang et al. [37] revealed divergent findings, compared to NC, patients with amnestic mild cognitive impairment (aMCI) exhibited increased ALFF/fALFF values in the bilateral parahippocampal gyrus/hippocampus and left inferior temporal gyrus. In contrast, patients with vascular mild cognitive impairment (vMCI) primarily showed decreased ALFF/fALFF values in the bilateral cuneus, left precuneus, left posterior cingulate cortex, and right cingulate gyrus. Our results indicated elevated fALFF in the hippocampus, insula, and inferior temporal gyrus in the MCA-S group, which aligns with Zhang’s findings but contradicts those of Xu’s. This discrepancy may arise from the potential inclusion of patients at an earlier preclinical stage of MCI, where chronic ischemia impairs cognition without manifesting overt symptoms [38]. The putamen and caudate nucleus, as components of the basal ganglia, are traditionally linked to motor control, habit learning, and reward processing. The caudate nucleus acts as a pivotal hub within the subcortical network, playing an important role in evaluating action-outcome contingency and orchestrating the planning and execution of tasks required for complex goals [39]. Wang et al. [40] observed that ischemic stroke patients exhibited lower fALFF values in the superior frontal gyrus and basal ganglia compared to healthy controls. Similarly, Han et al. [41] reported reduced fALFF values in the caudate nucleus and putamen among patients with post-stroke cognitive impairment. These findings differed from our results, likely because their study cohorts comprised patients with a history of stroke. The cerebellum plays a crucial role in a wide range of motor and cognitive tasks [42]. We found significantly increased fALFF in cerebellar Vermis_6 and cerebellar crus VIII, which may reflect enhanced sensorimotor and cognitive regulatory signals from the cerebral cortex to the cerebellum [42]. Furthermore, elevated fALFF values were present in several the cerebral hemispheres regions. The enhanced cortical activity may represent a compensatory response, possibly involving the recruitment of additional neurons to offset neuronal damage [43, 44]. Concurrently, prolonged and high-frequency neural inputs may also adaptively strengthen sensory processing pathways, consequently altering the functional activity patterns of the cerebellum.

Existing studies [15, 19] have a certain extent overlooked the temporal dimension. Our results indicated that vascular deficits not only reduce static signal intensity but also distort the temporal variability and stability of brain activity. The findings revealed significant differences in dfALFF values with CVR correction in the following regions: Vermis 7, the contralesional Cerebellum_8, ipsilesional Hippocampus, Frontal_Sup, Putamen, and Temporal_Inf. Notably, the dfALFF in the MCA-S group was significantly elevated. Group differences were present in the first five brain regions both without and with CVR correction, while the difference in the last region emerged only with CVR correction. We found both overlapping and distinct brain regions detected by sfALFF and dfALFF, which underscoring their mutual validation and complementarity [45, 46]. This divergence may also stem from residual vascular confounds interpreted as putative neuronal compensation or functional reorganization, a possibility requiring further investigation. The Vermis 7, identified as a novel region by dynamic metrics, is located in the anterior-superior cerebellar vermis. The cerebellum is integral to both motor and cognitive functions. The elevated dfALFF we observed in Vermis 7 may reflected enhanced sensorimotor and cognitive regulatory signals transmitted from the cerebral cortex to this region. Notably, Ding et al. [44] similarly reported increased fALFF values in the right cerebellar hemisphere and left vermis in patients with cognitive impairment associated with periventricular white matter lesions, interpreting as a compensatory response. Our results showed that dfALFF was sensitive to vascular constraints in a manner distinct from sfALFF. By correcting for CVR, we unmasked specific patterns of dynamic abnormality that were previously conflated with hemodynamic lag. This finding highlights that ignoring vascular factors can bias estimates of neural activity.

Although partial correlation analysis revealed no significant correlation between the scale scores and brain regions, the MCA-S group exhibited lower MMSE and MoCA scores compared to healthy controls, with a statistically significant difference in MMSE between the two groups. Previous study [47] had demonstrated intracranial large artery disease as a critical contributor to vascular cognitive impairment. Similarly, Hilal et al. [48] reported that intracranial arterial stenosis may lead to cognitive dysfunction via reduced cerebral perfusion. Consistent with our scale-based observations, the absence of a statistically significant difference in MoCA score may be related to the limited sample size or the potential compensatory effects of collateral circulation in some patients with intracranial stenosis, which could mitigate perfusion reduction. Future studies incorporating longitudinal follow-up data will further investigate these findings.

Our findings revealed the important impact of CVR correction in the rs-fMRI metrics in patients with cerebrovascular disease. Without CVR correction, significant group differences in sfALFF or dfALFF were observed in the ipsilesional Occipital_Mid, Parietal_Inf, Postcentral, and Temporal_Sup. However, these differences were no longer significant with CVR correction. Notably, all of these brain regions are primarily supplied by the MCA territory. This results suggested that the observed alterations in BOLD signal amplitude may have been influenced by abnormal CVR rather than solely reflecting neuronal dysfunction. The presence of between-group differences in CVR values within these regions further lends support to the interpretation.

In addtition, with CVR correction in our cohort, novel group differences emerged in several additional brain regions—including the contralesional Caudate and Temporal_Inf, ipsilesional Cerebelum_8 and Insula (static indicators), and the ipsilesional Temporal_Inf (dynamic indicators)—which had not shown significant differences without correction. These regions were identified only with CVR correction, indicating that hemodynamic noise in areas of vascular impairment likely obscured the underlying neural signals. Replicating these findings in other vascular diseases, such as moyamoya disease, may help clarify whether this pattern is specific to MCA-S patients or represents a more general phenomenon in cerebrovascular disease.

Previous studies on WMHs and cerebrovascular disease have documented the influence of cerebrovascular factors on BOLD signals. Ni et al. [15] reported in their investigation of cognitive impairments in WMH patients that more WMH-specific ALFF abnormalities could be detected when accounting for CVR effects. Furthermore, cerebrovascular factors have been shown to affect functional networks derived from resting-state BOLD data in studies of moyamoya disease and stroke patients [49, 50]. Wang et al. [19] showed that indicators affected by BOLD signal amplitude or intensity, such as ALFF, benefit more from intrinsic CVR correction. And this study represented the evaluation of the impact of intrinsic CVR on functional metrics across different spatial scales in stroke patients, while providing targeted correction recommendations for distinct functional indicators. Our study showed the necessity of CVR in fALFF and was a supplement to Wang et al.’s study. Our results aligned with these previous findings, further supporting the notion that vascular factors can confuse BOLD signals and highlighting the importance of CVR correction in distinguishing between neurovascular uncoupling and underlying neural dysfunction. These findings suggested that CVR correction should be implemented in future studies examining spontaneous neural activity (sfALFF/dfALFF) in patients with cerebrovascular diseases may enhance data reliability and interpretability.

There are several limitations in our research. Firstly, in this study, the internal CVR of patients with MCA-S was measured by the rs-fMRI method, but it was not verified by the clear CO2-targeted CVR method in previous studies. Liu et al. [27]demonstrated that rs-CVR maps show high spatial correlation with CO2-based CVR maps in healthy subjects(*r* = 0.88, *p* < 0.001) and patients with moyamoya(*r =* 0.71 ± 0.18). However, it is undeniable that this is a limitation of this study. Secondly, due to the relatively small sample size, we were unable to conduct subgroup analyses based on the lateralization of MCA stenosis (i.e., left vs. right). As a result, potential differences between left- and right-sided stenosis could not be fully explored. In future studies, we intend to increase the sample size and perform subgroup analyses to further investigate these effects. Thirdly, the results showed no correlations between the CVR-corrected fALFF metrics and neuropsychological scales. While CVR correction revealed additional brain regions with altered fALFF in MCA-S patients, the absence of brain–behavior associations tempers the functional interpretation of these findings. Finally, our study lacked the real-time blood pressure measurements on the specific day of the scan, preventing us from accurately assessing the impact of hypertension on CVR.

## Conclusion

In conclusion, MCA-S patients exhibited abnormal neuronal activity associated with both sfALFF and dfALFF. Differences were observed in the between-group comparisons of sfALFF and dfALFF with and without CVR correction, suggesting that the influence of CVR should be considered when evaluating neural activity in MCA-S patients. With CVR correction, the confounding effects of vascular factors on BOLD signals were partially mitigated, thereby enabling a more accurate reflection of underlying neural activity changes.

## Supplementary Information

Below is the link to the electronic supplementary material.

Supplementary Material 1

## Data Availability

The datasets used and/or analyzed during the current study are available from the corresponding author on reasonable request.

## References

[1] Li R, Lyu J, Hu R, Duan Q, Wang W, Bian X et al. Morphological study on lenticulostriate arteries in patients with middle cerebral artery stenosis at 7 T MRI. J Magn Reson Imaging. 2025;62(1):201–212. 10.1002/jmri.29693. 10.1002/jmri.29693 39781600

[2] Yang F, Shi W, Shi J, Zhang Y, Yin Y, Shi H, et al. Assessment of cerebrovascular reserve in unilateral middle cerebral artery stenosis using perfusion CT and CO2 inhalation tests. Int J Neurosci Taylor Francis. 2017;127:320–5. 10.1080/00207454.2016.1235044. 10.1080/00207454.2016.1235044 27619639

[3] Xu B, Li C, Guo Y, Xu K, Yang Y, Yu J. Current understanding of chronic total occlusion of the internal carotid artery. Biomed Rep. 2018;8:117–25. 10.3892/br.2017.1033. 29435269 10.3892/br.2017.1033 PMC5776422

[4] Duncombe J, Kitamura A, Hase Y, Ihara M, Kalaria RN, Horsburgh K. Chronic cerebral hypoperfusion: a key mechanism leading to vascular cognitive impairment and dementia. Closing the translational gap between rodent models and human vascular cognitive impairment and dementia. Clin Sci. 2017;131:2451–68. 10.1042/CS20160727. 10.1042/CS20160727 28963120

[5] Suri MFK, Zhou J, Qiao Y, Chu H, Qureshi AI, Mosley T, et al. Cognitive impairment and intracranial atherosclerotic stenosis in general population. Neurol Wolters Kluwer. 2018;90:e1240–7. 10.1212/WNL.0000000000005250. 10.1212/WNL.0000000000005250 PMC5890611 29523643

[6] Li J, Wang S, Li J, Fang Y, Wang Y, Zhang Y. Nomogram to predict cognitive impairment in patients with asymptomatic middle cerebral artery stenosis. IJGM Dove Press. 2023;16:1333–43. 10.2147/IJGM.S407728. 10.2147/IJGM.S407728 PMC10115204 37089137

[7] Zou Q-H, Zhu C-Z, Yang Y, Zuo X-N, Long X-Y, Cao Q-J, et al. An improved approach to detection of amplitude of low-frequency fluctuation (ALFF) for resting-state fMRI: Fractional ALFF. J Neurosci Methods. 2008;172:137–41. 10.1016/j.jneumeth.2008.04.012. 18501969 10.1016/j.jneumeth.2008.04.012 PMC3902859

[8] Cao X, Wang Z, Chen X, Liu Y, Abdoulaye IA, Ju S, et al. Changes in resting-state neural activity and nerve fibres in ischaemic stroke patients with hemiplegia. Brain Topogr. 2023;36:255–68. 10.1007/s10548-022-00937-6. 36604349 10.1007/s10548-022-00937-6

[9] Egorova N, Veldsman M, Cumming T, Brodtmann A. Fractional amplitude of low-frequency fluctuations (fALFF) in post-stroke depression. NeuroImage: Clin. 2017;16:116. 10.1016/j.nicl.2017.07.014. 28794972 10.1016/j.nicl.2017.07.014 PMC5537409

[10] Chen X, Onur OA, Richter N, Fassbender R, Gramespacher H, Befahr Q, et al. Concordance of intrinsic brain connectivity measures is disrupted in alzheimer’s disease. Brain Connectivity Mary Ann Liebert Inc publishers. 2023;13:344–55. 10.1089/brain.2020.0918. 10.1089/brain.2020.0918 34269605

[11] Faghiri A, Stephen JM, Wang Y-P, Wilson TW, Calhoun VD. Changing brain connectivity dynamics: From early childhood to adulthood. Hum Brain Mapp. 2017;39:1108. 10.1002/hbm.23896. 29205692 10.1002/hbm.23896 PMC5807176

[12] Dehaene S, Lau H, Kouider S. What is consciousness, and could machines have it? Science. 2017;358:486–92. 10.1126/science.aan8871. 29074769 10.1126/science.aan8871

[13] Liang L, Yuan Y, Wei Y, Yu B, Mai W, Duan G, et al. Recurrent and concurrent patterns of regional BOLD dynamics and functional connectivity dynamics in cognitive decline. Alzheimer’s Res Therapy. 2021;13:28. 10.1186/s13195-020-00764-6. 10.1186/s13195-020-00764-6 PMC7811744 33453729

[14] Tang Z, Liu T, Long J, Ren W, Liu Y, Li H, et al. Static and temporal dynamic changes in brain activity in patients with post-stroke balance dysfunction: A pilot resting state fMRI. Front NeuroSci. 2025;19:1558069. 10.3389/fnins.2025.1558069. 40182145 10.3389/fnins.2025.1558069 PMC11965596

[15] Ni L, Sun W, Yang D, Huang L, Shao P, Wang C, et al. The cerebrovascular reactivity-adjusted spontaneous brain activity abnormalities in white matter hyperintensities related cognitive impairment: A resting-state functional MRI study. J Alzheimers Dis. 2022;86:691–701. 10.3233/JAD-215216. 35124642 10.3233/JAD-215216

[16] Biswal BB, Kannurpatti SS, Rypma B. Hemodynamic scaling of fMRI-BOLD signal: Validation of low frequency spectral amplitude as a scalability factor. Magn Reson Imaging. 2007;25:1358–69. 10.1016/j.mri.2007.03.022. 17482411 10.1016/j.mri.2007.03.022 PMC2701471

[17] Halani S, Kwinta JB, Golestani AM, Khatamian YB, Chen JJ. Comparing cerebrovascular reactivity measured using BOLD and cerebral blood flow MRI: The effect of basal vascular tension on vasodilatory and vasoconstrictive reactivity. NeuroImage. 2015;110:110. 10.1016/j.neuroimage.2015.01.050. 25655446 10.1016/j.neuroimage.2015.01.050 PMC5167565

[18] Golestani AM, Kwinta JB, Strother SC, Khatamian YB, Chen JJ. The association between cerebrovascular reactivity and resting-state fMRI functional connectivity in healthy adults: The influence of basal carbon dioxide. NeuroImage. 2016;132:301–13. 10.1016/j.neuroimage.2016.02.051. 26908321 10.1016/j.neuroimage.2016.02.051 PMC5148617

[19] Wang L, Wu X, Song J, Fu Y, Ma Z, Wu X, et al. Unraveling the influences of hemodynamic lag and intrinsic cerebrovascular reactivity on functional metrics in ischemic stroke. NeuroImage. 2024;303:120920. 10.1016/j.neuroimage.2024.120920. 39521396 10.1016/j.neuroimage.2024.120920

[20] Song H, Zhang M, Wang Y, Yang L, Wang Y, Li Y. The impact of resilience on anxiety and depression among grass-roots civil servants in China. BMC Public Health. 2021;21:710. 10.1186/s12889-021-10710-2. 33849497 10.1186/s12889-021-10710-2 PMC8042932

[21] Chang Q, Xia Y, Bai S, Zhang X, Liu Y, Yao D, et al. Association between pittsburgh sleep quality index and depressive symptoms in Chinese resident physicians. Front Psychiatry. 2021;12:564815. 10.3389/fpsyt.2021.564815. 34149465 10.3389/fpsyt.2021.564815 PMC8206480

[22] Li T, Xu J, Wang L, Xu K, Chen W, Zhang L, et al. Functional network reorganization after endovascular thrombectomy in patients with anterior circulation stroke. NeuroImage: Clin. 2024;43:103648. 10.1016/j.nicl.2024.103648. 39067302 10.1016/j.nicl.2024.103648 PMC11332103

[23] Fazekas F, Chawluk J, Alavi A, Hurtig H, Zimmerman R. MR signal abnormalities at 1.5 T in alzheimer’s dementia and normal aging. Am J Roentgenol Am Roentgen Ray Soc. 1987;149:351–6. 10.2214/ajr.149.2.351. 10.2214/ajr.149.2.351 3496763

[24] van Laar PJ, Hendrikse J, Golay X, Lu H, van Osch MJP, van der Grond J. In vivo flow territory mapping of major brain feeding arteries. NeuroImage. 2006;29:136–44. 10.1016/j.neuroimage.2005.07.011. 16095923 10.1016/j.neuroimage.2005.07.011

[25] Tatu L, Moulin T, Vuillier F, Bogousslavsky J. Arterial territories of the human brain. Front Neurol Neurosci. 2012;30:99–110. 10.1159/000333602. 10.1159/000333602 22377874

[26] Tang X, Wang L, Feng Q, Hu H, Zhu Y, Liao Z, et al. Resting-state functional magnetic resonance imaging study on cerebrovascular reactivity changes in the precuneus of Alzheimer’s disease and mild cognitive impairment patients. Sci Rep. 2025;15:363. 10.1038/s41598-024-82769-x. 39747269 10.1038/s41598-024-82769-x PMC11696737

[27] Liu P, Li Y, Pinho M, Park DC, Welch BG, Lu H. Cerebrovascular reactivity mapping without gas challenges. NeuroImage. 2017;146:320–6. 10.1016/j.neuroimage.2016.11.054. 27888058 10.1016/j.neuroimage.2016.11.054 PMC5321860

[28] Hu C, Wang L, Ge X, Han Z, Zhang X, Du X, et al. Frequency-dependent and temporal variability of low-frequency fluctuations in patients with primary sjögren’s syndrome. Lupus Sci Med. 2025;12:e001655. 10.1136/lupus-2025-001655. 40962361 10.1136/lupus-2025-001655 PMC12443196

[29] Wang X, Wang C, Liu J, Guo J, Miao P, Wei Y, et al. Altered static and dynamic spontaneous neural activity in patients with ischemic pontine stroke. Front Neurosci. 2023;17:1131062. 10.3389/fnins.2023.1131062. 37008224 10.3389/fnins.2023.1131062 PMC10060846

[30] Niu X, Gao X, Zhang M, Dang J, Sun J, Lang Y, et al. Static and dynamic changes of intrinsic brain local connectivity in internet gaming disorder. BMC Psychiatry. 2023;23:578. 10.1186/s12888-023-05009-y. 37558974 10.1186/s12888-023-05009-y PMC10410779

[31] Cui Q, Sheng W, Chen Y, Pang Y, Lu F, Tang Q, et al. Dynamic changes of amplitude of low-frequency fluctuations in patients with generalized anxiety disorder. Hum Brain Mapp. 2020;41:1667–76. 10.1002/hbm.24902. 31849148 10.1002/hbm.24902 PMC7267950

[32] N T-M BL, F DP, O E C. Automated anatomical labeling of activations in SPM using a macroscopic anatomical parcellation of the MNI MRI single-subject brain. Neuroimage [Internet] Neuroimage. 2002. 10.1006/nimg.2001.0978. [cited 2026 Mar 10];15. 10.1006/nimg.2001.0978 11771995

[33] Ni L, Wen J, Zhang LJ, Zhu T, Qi R, Xu Q, et al. Aberrant default-mode functional connectivity in patients with end-stage renal disease: A resting-state functional MR imaging study. Radiol [Internet] Radiological Soc North Am. 2014. 10.1148/radiol.13130816. [cited 2025 June 9]. 10.1148/radiol.13130816 24484062

[34] Lu H, Gu Z, Xing W, Han S, Wu J, Zhou H, et al. Alterations of default mode functional connectivity in individuals with end-stage renal disease and mild cognitive impairment. BMC Nephrol. 2019;20:246. 10.1186/s12882-019-1435-6. 31277581 10.1186/s12882-019-1435-6 PMC6612101

[35] Huang S, Wen X, Liu Z, Li C, He Y, Liang J, et al. Distinguishing functional and structural MRI abnormalities between bipolar and unipolar depression. Front Psychiatry. 2023;14:1343195. 10.3389/fpsyt.2023.1343195. 38169701 10.3389/fpsyt.2023.1343195 PMC10758430

[36] Xu S, Fan Y, Mao C, Hu Z, Yang Z, Qu L, et al. Multimodal magnetic resonance imaging analysis of early mild cognitive impairment. J Alzheimers Dis. 2025;104:1013–27. 10.1177/13872877251321187. 40033775 10.1177/13872877251321187

[37] Zhang X, Xue C, Cao X, Yuan Q, Qi W, Xu W, et al. Altered patterns of amplitude of low-frequency fluctuations and fractional amplitude of low-frequency fluctuations between amnestic and vascular mild cognitive impairment: An ALE-based comparative meta-analysis. Front Aging Neurosci. 2021;13:711023. 10.3389/fnagi.2021.711023. 34531735 10.3389/fnagi.2021.711023 PMC8438295

[38] Huang Z, Xia X, Guan S, Gong G, Luo Y, Shi L, et al. Neuroimaging anomalies in asymptomatic middle cerebral artery steno-occlusive disease with normal-appearing white matter. Front Neurol. 2023;14:1206786. 10.3389/fneur.2023.1206786. 37693758 10.3389/fneur.2023.1206786 PMC10484479

[39] Duan LY, Horst NK, Cranmore SAW, Horiguchi N, Cardinal RN, Roberts AC, et al. Controlling one’s world: Identification of sub-regions of primate PFC underlying goal-directed behavior. Neuron. 2021;109:2485–e24985. 10.1016/j.neuron.2021.06.003. 34171290 10.1016/j.neuron.2021.06.003 PMC8346232

[40] Wang S, Rao B, Chen L, Chen Z, Fang P, Miao G, et al. Using fractional amplitude of low-frequency fluctuations and functional connectivity in patients with post-stroke cognitive impairment for a simulated stimulation program. Front Aging Neurosci. 2021;13:724267. 10.3389/fnagi.2021.724267. 34483891 10.3389/fnagi.2021.724267 PMC8414996

[41] Han K, Dong L, Liao X, Long J, Chen J, Lu H, et al. Alterations in brain function in patients with post-stroke cognitive impairment: A resting-state functional magnetic resonance imaging study. Front Aging Neurosci. 2025;17:1501082. 10.3389/fnagi.2025.1501082. 40046780 10.3389/fnagi.2025.1501082 PMC11880027

[42] King M, Hernandez-Castillo CR, Poldrack RA, Ivry RB, Diedrichsen J. Functional boundaries in the human cerebellum revealed by a multi-domain task battery. Nat Neurosci. 2019;22:1371–8. 10.1038/s41593-019-0436-x. 31285616 10.1038/s41593-019-0436-x PMC8312478

[43] Li C, Zheng J, Wang J, Gui L, Li C. An fMRI stroop task study of prefrontal cortical function in normal aging, mild cognitive impairment, and alzheimer’s disease. Curr Alzheimer Res. 2009;6:525–30. 10.2174/156720509790147142. 19747163 10.2174/156720509790147142

[44] Ding X, Wu J, Zhou Z, Zheng J. Specific locations within the white matter and cortex are involved in the cognitive impairments associated with periventricular white matter lesions (PWMLs). Behav Brain Res. 2015;289:9–18. 10.1016/j.bbr.2015.04.021. 25899094 10.1016/j.bbr.2015.04.021

[45] Faghiri A, Stephen JM, Wang Y-P, Wilson TW, Calhoun VD. Changing brain connectivity dynamics: From early childhood to adulthood. Hum Brain Mapp. 2018;39:1108–17. 10.1002/hbm.23896. 29205692 10.1002/hbm.23896 PMC5807176

[46] Ge X, Wang L, Yan J, Pan L, Ye H, Zhu X, et al. Altered brain function in classical trigeminal neuralgia patients: ALFF, ReHo, and DC static- and dynamic-frequency study. Cereb Cortex. 2024;34:bhad455. 10.1093/cercor/bhad455. 38012118 10.1093/cercor/bhad455

[47] Arenillas JF, Dieleman N, Bos D. Intracranial arterial wall imaging: Techniques, clinical applicability, and future perspectives. Int J Stroke. 2019;14:564–73. 10.1177/1747493019840942. 30982434 10.1177/1747493019840942

[48] Hilal S, Mutsaerts HJMM, Ferro DA, Petr J, Kuijf HJ, Biessels GJ, et al. The effects of intracranial stenosis on cerebral perfusion and cognitive performance. J Alzheimers Dis. 2021;79:1369–80. 10.3233/JAD-201131. 33427743 10.3233/JAD-201131

[49] Christen T, Jahanian H, Ni WW, Qiu D, Moseley ME, Zaharchuk G. Noncontrast mapping of arterial delay and functional connectivity using resting-state functional MRI: A study in moyamoya patients. J Magn Reson Imaging. 2015;41:424–30. 10.1002/jmri.24558. 24419985 10.1002/jmri.24558 PMC4096618

[50] Siegel JS, Snyder AZ, Ramsey L, Shulman GL, Corbetta M. The effects of hemodynamic lag on functional connectivity and behavior after stroke. J Cereb Blood Flow Metab. 2016;36:2162–76. 10.1177/0271678X15614846. 26661223 10.1177/0271678X15614846 PMC5363662

