# Correction to: The Cerebrovascular Reactivity Adjusted Fractional Amplitude of Low-Frequency Fluctuations Abnormalities in Middle Cerebral Artery Stenosis and Occlusive Disease

**DOI:** 10.1007/s12975-026-01466-1

**Published:** 2026-07-01

**Authors:** Liqing Zhang, Luoyu Wang, Xue Tang, Yidi Zhu, Rong Wang, Zhongxiang Ding

**Affiliations:** 1 Department of Radiology, Affiliated Hangzhou First People’s Hospital, School of Medicine, Westlake University, Hangzhou, 310006 China; 2 School of Biomedical Engineering, ShanghaiTech University, Shanghai, 201210 China; 3 Center for Cognition and Brain Disorders, The Affiliated Hospital of Hangzhou Normal University, Hangzhou, 311121 China; 4 School of Medical Imaging, Hangzhou Medical College, Hangzhou, 310006 China

**Correction to: Translational Stroke Research (2026) 17:38**

**https://doi.org/10.1007/s12975-026-01430-z**

In the originally published version of this article, the corresponding author designation (the envelope icon) was inadvertently omitted for co-corresponding author Zhongxiang Ding.

Please note that both Luoyu Wang and Zhongxiang Ding are co-corresponding authors for this article.

The original article has been updated.

