# Supplementary material for: The Cerebrovascular Reactivity Adjusted Fractional Amplitude of Low-Frequency Fluctuations Abnormalities in Middle Cerebral Artery Stenosis and Occlusive Disease
Source: Transl Stroke Res. 2026 Apr 1;17(2):38. doi: 10.1007/s12975-026-01430-z (PMC13043580; doi:10.1007/s12975-026-01430-z)
Supplement: Supplementary file 1 — Supplementary Material 1 [file 12975_2026_1430_MOESM1_ESM.docx]

**
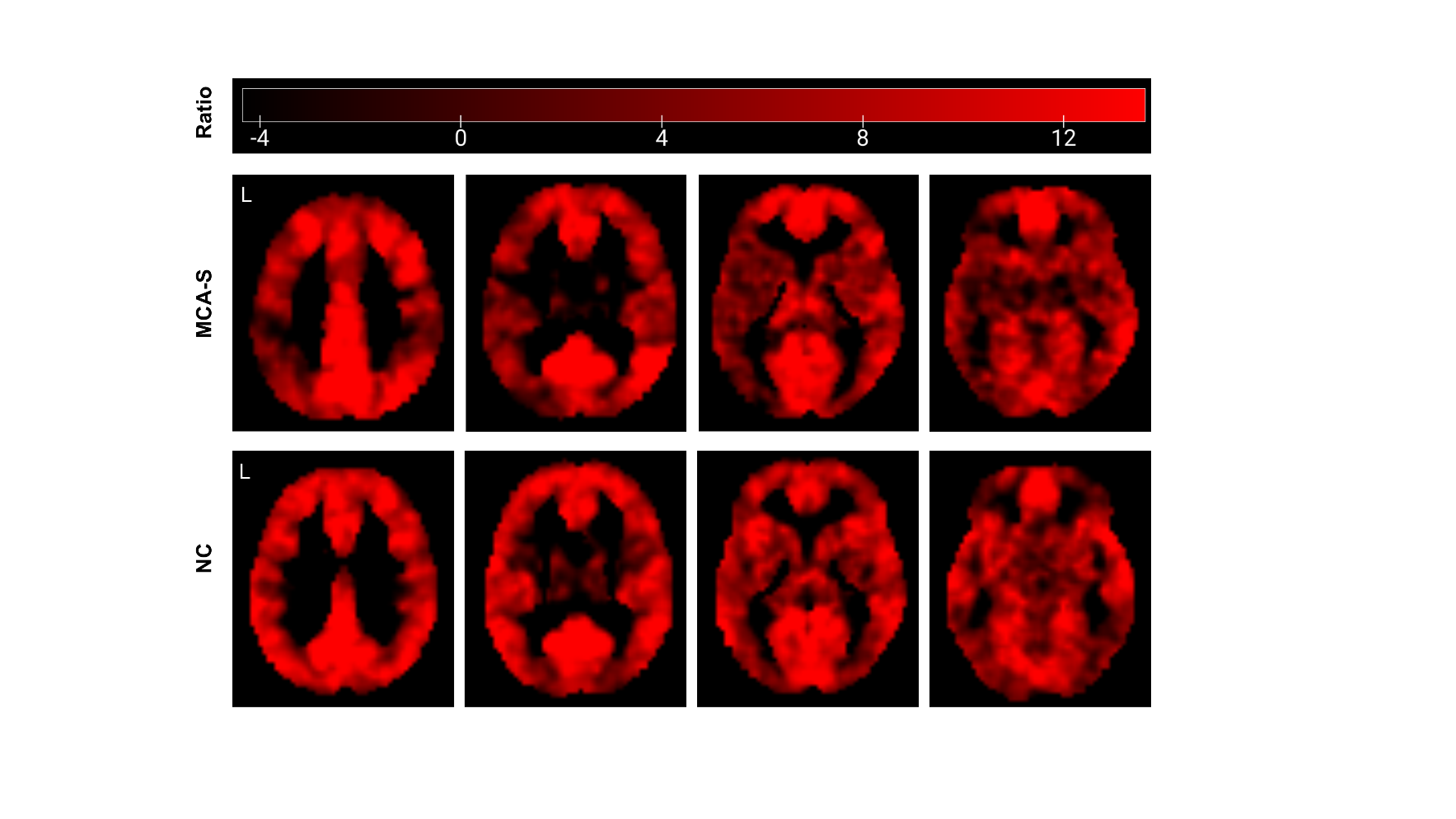
**

**Figure S1. The averaged CVR maps obtained from the MCA-S and NC groups.** Row 1: CVR maps using resting-state scan of MCA-S patients. Row 2: CVR maps using resting-state scan of NC. CVR, Cerebrovascular reactivity; MCA-S, Middle cerebral artery stenosis; NC, Normal control.


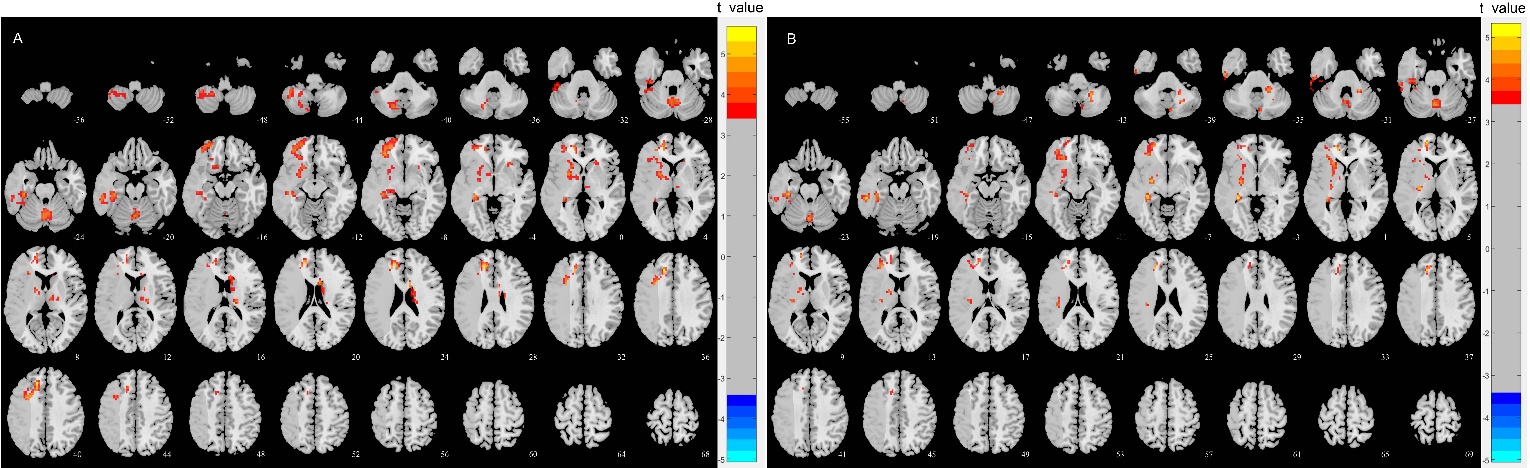


**Figure S2. Altered brain regions in sfALFF and dfALFF MCA-S patients compared to NC with CVR correction combined visualization of the middle cerebral artery territory (**Age, gender, education level and hypertension were included as covariates.)**.** **(A) sfALFF, (B) dfALFF.** CVR, Cerebrovascular reactivity; dfALFF, Dynamic amplitude of low frequency fluctuation; MCA-S, Middle cerebral artery stenosis; NC, Normal control; sfALFF, Static amplitude of low frequency fluctuation.

**
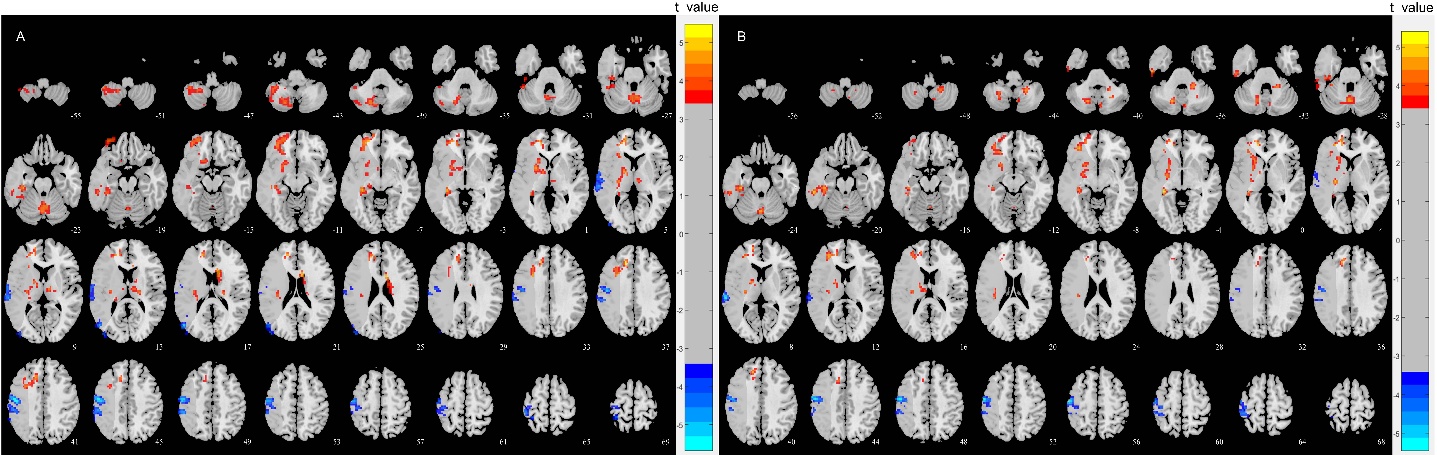
**

**Figure S3. Altered brain regions in sfALFF and dfALFF MCA-S patients compared to NC without CVR correction combined visualization of the middle cerebral artery territory (**Age, gender, education level and hypertension were included as covariates.**).** **(A) sfALFF, (B) dfALFF.** CVR, Cerebrovascular reactivity; dfALFF, Dynamic amplitude of low frequency fluctuation; MCA-S, Middle cerebral artery stenosis; NC, Normal control; sfALFF, Static amplitude of low frequency fluctuation.

**Table S1. Brain regions of significant differences in sfALFF, dfALFF with CVR correction between MCA-S and NC groups*.**

| Brain regions | | Peak MNI coordinates | | | Cluster size(voxels) | Peak intensity | *P* |
| --- | --- | --- | --- | --- | --- | --- | --- |
|  |  | X | Y | Z |  |  |  |
| sfALFF | Hippocampus_IL | -33 | -30 | -6 | 43 | 4.9419 | <0.001 |
|  | Vermis_6 | -3 | -66 | -24 | 32 | 4.3579 | <0.001 |
|  | Frontal_Sup_IL | -18 | 39 | 27 | 108 | 5.6666 | <0.001 |
|  | Putamen_IL | -24 | 6 | 0 | 58 | 4.5313 | <0.001 |
|  | Cerebelum_8_ IL | -39 | -42 | -45 | 62 | 3.9766 | <0.001 |
|  | Temporal_Inf_CON | 48 | -39 | -15 | 39 | 4.2651 | <0.001 |
|  | Caudate_CON | 9 | 9 | 21 | 31 | 4.7936 | <0.001 |
| dfALFF | Cerebelum_8_CON | 21 | -48 | -42 | 16 | 4.5489 | <0.001 |
|  | Hippocampus_IL | -33 | -33 | -3 | 28 | 5.0432 | <0.001 |
|  | Vermis_7 | -3 | -66 | -27 | 10 | 4.3049 | <0.001 |
|  | Frontal_Sup_IL | -15 | 57 | 6 | 61 | 5.1204 | <0.001 |

* Age, gender, education level and hypertension were included as covariates. Caudate, Caudate nucleus; Cerebelum_8, Cerebellum Crus VIII‌; CON, Contralateral; CVR, Cerebrovascular reactivity; dfALFF, Dynamic amplitude of low frequency fluctuation; Frontal_Sup, Superior Frontal gyrus; IL, ipsilateral; MCA-S, Middle cerebral artery stenosis; MNI, Montreal neurological institute; NC, Normal control; Putamen, Lenticular nucleus putamen; sfALFF, Static amplitude of low frequency fluctuation; Temporal_Inf, Inferior Temporal gyrus; Vermis_6, Cerebellar Vermis VI;Vermis_7, Cerebellar Vermis VII.

**Table S2. Brain regions of significant differences in sfALFF, dfALFF without CVR correction between MCA-S and NC groups*.**

| Brain regions | | Peak MNI coordinates | | | Cluster size(voxels) | Peak intensity | *P* |
| --- | --- | --- | --- | --- | --- | --- | --- |
|  |  | X | Y | Z |  |  |  |
| sfALFF | Hippocampus_IL | -33 | -30 | -6 | 45 | 4.9738 | <0.001 |
|  | Vermis_6 | -3 | -63 | -24 | 33 | 4.3244 | <0.001 |
|  | Frontal_Sup_IL | -15 | 33 | 36 | 96 | 5.3655 | <0.001 |
|  | Putamen_IL | -24 | 6 | 0 | 79 | 4.7795 | <0.001 |
|  | Occipital_Mid_IL | -51 | -72 | 15 | 12 | -5.5453 | <0.001 |
|  | Thalamus_CON | 9 | 9 | 21 | 42 | 4.9332 | <0.001 |
|  | Postcentral_IL | -51 | -12 | 42 | 278 | -5.4519 | <0.001 |
| dfALFF | Cerebelum_8_IL | -18 | -66 | -36 | 37 | 4.5041 | <0.001 |
|  | Cerebelum_8_CON | 21 | -48 | -42 | 18 | 4.5790 | <0.001 |
|  | Hippocampus_IL | -33 | -33 | -3 | 28 | 5.0845 | <0.001 |
|  | Frontal_Sup_IL | -15 | 57 | 6 | 58 | 5.1458 | <0.001 |
|  | Temporal_Sup_IL | -63 | -27 | 9 | 53 | -5.0131 | <0.001 |
|  | Postcentral_IL | -54 | -12 | 45 | 201 | -5.3621 | <0.001 |

*Age, gender, education level and hypertension were included as covariates. Cerebelum_8, Cerebellum Crus VIII‌; CON, Contralateral; CVR, Cerebrovascular reactivity; dfALFF, Dynamic amplitude of low frequency fluctuation; Frontal_Sup, Superior Frontal gyrus; IL, ipsilateral; MCA-S, Middle cerebral artery stenosis; MNI, Montreal neurological institute; NC, Normal control; Occipital_Mid, Middle Occipital gyrus; Postcentral, Postcentral gyrus; sfALFF, Static amplitude of low frequency fluctuation; Temporal_Sup, Superior temporal gyru; Vermis_6, Cerebellar Vermis VI.
